# Development of an Antibacterial Coating Based on PVP–PEG Fibers Incorporating Silver Nanoparticles and a Method for Its Application to Skin

**DOI:** 10.3390/polym18091117

**Published:** 2026-04-30

**Authors:** Elizaveta Mokhova, Natalia Menshutina, Sergei Kalenov, Svetlana Evdokimova, Anastasiya Shirokikh, Ksenia Serkina

**Affiliations:** 1 The Department of Chemical and Pharmaceutical Engineering, Mendeleev University of Chemical Technology of Russia (MUCTR), Moscow 125047, Russia; mokhova.e.k@muctr.ru; 2 The Department of Biotechnology, Mendeleev University of Chemical Technology of Russia (MUCTR), Moscow 125047, Russia; kalenov.s.v@muctr.ru (S.K.);; 3 The Department of Nanomaterials and Nanotechnologies, Mendeleev University of Chemical Technology of Russia (MUCTR), Moscow 125047, Russia; shirokikh.a.d@muctr.ru; 4 The Department of Chemistry and Technology of Crystals, Mendeleev University of Chemical Technology of Russia (MUCTR), Moscow 125047, Russia; serkina.k.s@muctr.ru

**Keywords:** micro- and nanofibers, polyvinylpyrrolidone, polyethylene glycol, silver nanoparticles, electrospinning, portable device

## Abstract

This article presents the results of the development of an antibacterial coating based on polyvinylpyrrolidone (PVP) and polyethylene glycol (PEG) fibers with embedded silver nanoparticles. Silver nanoparticles were synthesized via the use of PEG, which acts as a reducing agent for Ag^+^ ions and a stabilizer for the colloidal system. The resulting sols were pink, dark purple, and orange color. The viscosity of the compositions, which increased with increasing PEG and AgNO_3_ concentrations, was studied. The sizes of the synthesized silver nanoparticles were determined via dynamic light scattering. For all compositions, monomodal particle size distributions were obtained with characteristic sizes of 50.75, 58.73, 13.54 and 28.21 nm. The highest ζ-potential value for the silver nanoparticles was −15.5 mV, indicating their stability. The electrical conductivity of the compositions increased with increasing molar concentration of AgNO_3_. The resulting PVP-PEG compositions with silver nanoparticles demonstrated resistance to pathogenic bacteria such as *Staphylococcus aureus* and *Escherichia coli*. A portable electrospinning device was developed at the Mendeleev University of Chemical Technology of Russia to apply the compositions to the skin and form a protective coating of PVP-PEG fibers with an antibacterial effect. Fiber formation was confirmed by scanning electron microscopy. The incorporation of silver into the fiber structure was confirmed by the results of elemental analysis and surface mapping of the samples.

## 1. Introduction

Currently, regenerative medicine is a rapidly developing field of science aimed at restoring damaged or diseased cells and tissues [1,2]. One area of regenerative medicine is the development of new functional biomedical materials for the treatment or restoration of damaged skin or deeper tissues [3]. The skin performs an important function in the human body, protecting it from external influences and participating in thermoregulation and metabolic processes [4]. Various types of mechanical, thermal, and chemical influences or internal processes occurring in the human body can lead to dysfunction of this organ and, in the most severe cases, to tissue degradation and amputation of the limbs [5]. The most common and serious types of damage to the skin include superficial wounds, burns and chronic wounds [6,7]. Superficial wounds typically involve the epidermis and upper dermis and include abrasions, cuts, and first-degree burns, which are accompanied by minimal bleeding, redness, and inflammation [8]. These types of wounds require cleaning the affected area, maintaining normal fluid balance and gas exchange to ensure effective healing, and monitoring for signs of infection. Chronic wounds take a long time to heal (more than one to three months) or do not heal at all and are often accompanied by infectious complications [9]. To prevent infection, antibiotics are typically administered systemically, with doses often exceeding the recommended values, which leads to the development of resistance to specific types of antibiotics in patients [10]. To address this important clinical problem, the scientific community is looking for new methods for the local delivery of drugs or antibacterial agents to the wound site to complement existing therapies [11].

In recent years, polymer films, which are thin, flexible and transparent dressing materials, have been developed in the field of wound treatment, including burns. Polymer films can be used directly for application as wound healing dressings [12,13], as can transdermal systems for delivering drugs through the upper layers of the skin into blood vessels [14]. However, the main disadvantages of film materials are the labor intensity of their creation, the scaling of production technology and the difficulty in handling the final product [15]. In addition to film materials for wound treatment, innovative nonwoven polymer materials based on micro- and nanofibers have begun to be developed [16,17]. Polymer nanofiber dressings are intended not only to protect wounds from environmental influences but also to stimulate the regeneration process of damaged tissue [18,19]. These dressings have a high specific surface area and provide gas permeability for the normal wound healing process while blocking the access of microorganisms. To reduce the regeneration time of damaged tissues, nanofibers with the addition of growth factors that stimulate the growth and development of cells are used [20]. However, to address the issue of resistance, dressings are modified not with antibiotics but with nanoparticles of various metals, such as silver, gold, copper, zinc oxide, and titanium dioxide, which have antibacterial properties [21]. These materials affect bacterial cells mainly by releasing metal ions, which additionally increase the production of reactive oxygen species and affect the metabolism of bacteria, leading to destruction of their cell wall [22]. The main causative agents of purulent-inflammatory processes in the wound area are the Gram-positive bacteria *Staphylococcus aureus* and *Staphylococcus epidermidis*, and when the wound is contaminated or the immune system is weakened, Gram-negative bacteria, such as *Escherichia coli*, *Proteus mirabilis* and *Enterobacter*, are present [23]. Metal nanoparticles effectively combat pathogens while reducing the development of resistance [24].

In addition to the development of nonwoven materials, the method of applying fibers to damaged skin remains a significant challenge. The application of a dressing to a wound often requires fingertip contact with the material surface during application, which can lead to contamination of the wound-adjacent surface beneath the dressing. Therefore, maintaining dressing sterility during application is an important factor in dressing development. To address this issue, a variety of portable electrospinning devices have been developed that can form fibers directly on damaged skin [25,26,27]. The paper [25] presents a hand-held, gun-type electrospinning device with dimensions of 12.5 × 4 × 14.1 cm. The device’s body is made of photopolymer resin, and the main operating elements—a high-voltage unit, a lithium battery, and a power button—are hidden inside the front of the body. This device can operate at voltages up to 10 kV. The disadvantages of the device include: 1—the body and the regulator of manual extrusion are made of photopolymer resin, since parts made of photopolymer resin are characterized by increased fragility; 2—the design does not include a front cover, which should hide all electrical components; 3—the lithium battery does not have a socket for recharging; 4—the handle of the device can be uncomfortable to use, due to limited dimensions, it may not be suitable for different users (the size of palms in different people can vary greatly). The article [26] presents various designs of portable electrospinning devices. For example, a description is given of an electrospinning gun from Qingdao Junada Technology Co., Ltd. (Qingdao, China), which consists of a pistol-type handle with a high-voltage unit and a separate air duct that provides directed deposition of fibers. However, such a design cannot be classified as a portable device, since the air duct unit is too large and heavy. In [28], a method for miniaturizing the above-described design with a directed air flow is proposed. The described device includes both a miniature high-precision microsyringe pump and a miniature high-voltage power source capable of generating up to 33 kV at 10 W. Despite the reduction in structural elements, the device [28] lacks a well-designed ergonomic housing, and a power of 10 W will lead to rapid overheating of the device. A study [27] presented a device from NANO Fiberlabs (Foshan, China) used to produce dressings. This device operates at 10 kV and a spray distance of 15 cm. The disadvantages of the device include: 1—the operating voltage is 10 kV, as lower voltages can lead to a reduction in spray distance depending on the compositions used; 2—the device’s design requires vertical placement during use, which can lead to solution dripping due to gravity. Finding the most optimal designs in terms of functionality and ergonomics is an important task.

The aim of this study was to develop an antibacterial coating based on fibers of biocompatible PVP and PEG polymers with embedded silver nanoparticles, to study the properties of the synthesized nanoparticles and fiber coatings, and to develop a portable electrospinning device capable of effectively applying fibers to the skin. It should be noted that the scientific literature mainly describes fibers obtained either separately from PVP and PEG or from their combination [29,30,31,32]; methods for the synthesis and stabilization of silver nanoparticles using PEG and PVP in solution have been separately considered [33,34]. However, there are no works describing the production of fibers from a combination of PVP-PEG with synthesized silver nanoparticles and investigating their structural and antibacterial properties, which emphasizes the significance of this study.

## 2. Materials and Methods

### 2.1. Materials

The following chemical reagents and materials were used in this work. Polyvinylpyrrolidone (PVP) grade Kollidon-90 with a molecular weight of 360 kDa (Sigma-Aldrich, St. Louis, MO, USA) was used. Polyethylene glycol 6000 (PEG 6000) with a molecular weight of 6 kDa was used (Kepec Chemische Fabrik GmbH, Siegburg, Germany). Silver nitrate (AgNO_3_) was chemically pure (Lenreaktiv, St. Petersburg, Russia). Isopropyl alcohol (IPA) with an active substance content of 99.7% (Kemstor, Moscow, Russia) was used. Distilled water.

### 2.2. Preparation of Solutions of PVP, PEG and PVP-PEG Mixtures

A 60% IPA was used to dissolve the PVP, PEG, and PVP-PEG mixtures. Table 1 presents the specifications of the studied formulations.

Each of the solutions was stirred on an IKA RCT magnetic stirrer (IKA, Staufen im Breisgau, Germany) at 700 rpm for 1 h until the polymers were completely dissolved.

### 2.3. Method for Synthesizing Silver Nanoparticles

**Figure 1:**
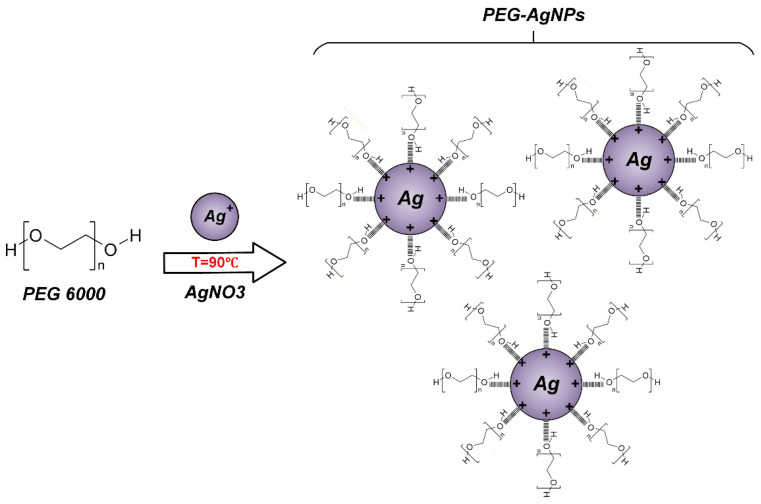
The mechanism of the reduction of Ag^+^ by PEG to AgNPs with subsequent stabilization of the colloidal system.

The method for synthesizing silver nanoparticles (AgNPs) is based on the reduction of silver ions Ag^+^ with PEG. PEG is a multifunctional polymer capable of performing dual roles in the synthesis of silver nanoparticles, simultaneously acting as a reducing agent and a colloidal system stabilizer. This is due to the presence of hydroxyl groups in its structure, which are capable of reducing Ag^+^ to metallic Ag^0^ under relatively mild synthesis conditions. Figure 1 shows the mechanism for producing silver nanoparticles.

To obtain PVP-PEG compositions with AgNPs, PVP and PEG polymer samples weighed on an AND HR-100AG analytical balance (AND, Tokyo, Japan) were dissolved in 60% IPA until homogeneous solutions were obtained. AgNO_3_, which was previously dissolved in 60% IPA, was then added to the resulting solutions. Table 2 presents the specifications for the final PVP-PEG compositions with embedded AgNPs.

Each solution was stirred on an IKA RCT magnetic stirrer (IKA, Staufen im Breisgau, Germany) at 1200 rpm and a core temperature of 90 °C for 2 h. The core temperature was monitored via an immersion thermocouple. The resulting solutions were cooled to room temperature and transferred to test tubes for subsequent use.

### 2.4. Measuring the Viscosity of the Initial Solutions

Viscosity studies were conducted for PVP-PEG-1, PVP-PEG-2, PVP-PEG-1-Ag1, PVP-PEG-2-Ag1, PVP-PEG-1-Ag2 and PVP-PEG-2-Ag2 solutions via an Anton Paar SmartPave 102e rotational rheometer (Anton Paar, Graz, Austria). Measurements were performed at a constant shear rate of 0.1 s^−1^ and a temperature of 25 °C. The number of measurement points for each solution was 20. The analytical study was conducted at the Department of Chemical and Pharmaceutical Engineering of the MUCTR.

### 2.5. Determination of Silver Concentration in the Studied Compositions

Silver concentrations in the compounds were determined using inductively coupled plasma optical emission spectrometry (ICP-OES). PlasmaQuant PQ 9100 equipment (Analytik Jena, Jena, Germany) was used for the study. The analytical study was conducted at the MUCTR Shared Use Center.

### 2.6. Method for Determining the Diameter and ζ-Potential of Silver Nanoparticles

The diameter of the synthesized silver nanoparticles was determined via dynamic light scattering using a Zetasizer Nano ZS instrument (Malvern Instruments, Malvern, UK) equipped with a helium-neon laser (λ = 633 nm). Scattered light was recorded at an angle of 173°. Nanoparticle sizes were measured without dilution of the systems. Each measurement included an average of 14 scans. Measurements were performed at a temperature of 25 °C. To calculate the average nanoparticle diameter, the results of at least five measurements performed for each sample were used.

The ζ-potentials of the AgNPs were calculated on the basis of their electrophoretic mobility, which was measured via a Zetasizer Nano ZS analyzer. Measurements were performed at 25 °C. Each sample was analyzed at least five times, after which the average value was calculated. The analytical studies were conducted at the Department of Nanomaterials and Nanotechnology of the MUCTR.

### 2.7. Methodology for Conducting UV Spectroscopy

A UNICO SQ-2800 instrument (United Products and Instruments, Dayton, NJ, USA) was used for UV spectroscopy. Before the main measurement, a solvent baseline (60% IPA) was constructed. The following stock solutions were subsequently analyzed: PVP-1, PEG-1, PEG-2, PVP-PEG-1, PVP-PEG-2, PVP-PEG-1-Ag1, PVP-PEG-2-Ag1, PVP-PEG-1-Ag2, and PVP-PEG-2-Ag2. Each solution was sequentially poured into 9 mL cuvettes and placed in the spectrophotometer opposite the light source. Measurements were performed in the spectral range from 190–1000 nm. The analytical study was conducted at the Department of Chemical and Pharmaceutical Engineering of the MUCTR.

### 2.8. Measurement of Electrical Conductivity of Initial Solutions

A portable TDS&EC conductivity meter (Kelly Union Electronics, Hong Kong, China) with a measurement resolution of 1 μS/cm was used to measure the electrical conductivity of the PVP-PEG-1, PVP-PEG-2, PVP-PEG-1-Ag1, PVP-PEG-2-Ag1, and PVP-PEG-2-Ag2 solutions. The measuring device was immersed in 20 mL of the analyzed solutions so that the electrodes were completely immersed. Three measurements were taken for each solution, after which the obtained values were recorded. The measurements were performed at the Department of Chemical and Pharmaceutical Engineering of the MUCTR.

### 2.9. Development of a Design for Portable Electrospinning Device

**Figure 2:**
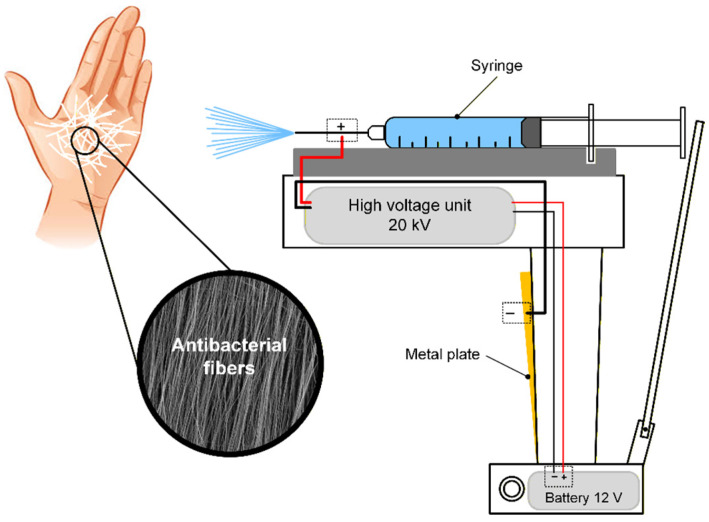
Schematic diagram of the portable electrospinning device and the process of forming a fiber coating.

A portable electrospinning device was developed for the rapid application of PVP-PEG and PVP-PEG with embedded AgNPs in the skin. This device enables the efficient formation and application of polymer-based antibacterial compounds to damaged skin areas. Figure 2 shows a scheme of the device and the fiber coating formation process.

Electrospinning operates by creating a potential difference between the tip of the needle, through which the polymer solution is injected, and a grounded collector, on which the resulting nanofiber is deposited. In the portable electrospinning device, all the main components are concealed within a compact housing: a 12 V Li-Ion battery (Aleaivy, Shenzhen, China), a 20 kV high-voltage unit with a 5 W nominal power, 12 V input voltage and 0.1 A current consumption (no load) (Diymore, Hong Kong, China), and a module housing a 5 mL syringe. An electrode, responsible for the negative charge, is connected from the high-voltage unit to a metal plate attached to the handle. This allows the charge to be transferred through the hand of the person holding the handle of the portable electrospinning device, preventing charge accumulation.

The device design was completed via Autodesk Inventor Professional 2022. The device body was 3D printed via fused deposition technology on a Bambu Lab P2S Combo (Bambu Lab, Shenzhen, China) using PLA plastic.

### 2.10. Method for Obtaining Fiber Materials Based on PVP-PEG and PVP-PEG with Embedded AgNPs

Fiber samples were obtained as follows. Solutions of PVP-PEG-1, PVP-PEG-2, PVP-PEG-1-Ag1, PVP-PEG-2-Ag1, PVP-PEG-1-Ag2, and PVP-PEG-2-Ag2 were drawn into a syringe (2.5 mL each). The syringe was then installed in a portable electrospinning device. The device was held with one hand so that the hand touched the metal plate on the handle, whereas the other hand held a 15 × 15 cm aluminum foil at a distance of 20 cm from the tip of the needle to collect the fibers. The inner diameter of the needle was 1 mm. The process was carried out at a voltage of 20 kV.

### 2.11. Scanning Electron Microscopy

A JEOL JSM-IT500 scanning electron microscope (JEOL, Akishima, Japan) was used to analyze the structures of the PVP-PEG-1, PVP-PEG-2, PVP-PEG-1-Ag1, PVP-PEG-2-Ag1, PVP-PEG-1-Ag2, and PVP-PEG-2-Ag2 fiber samples. The analytical study was conducted at M.V. Lomonosov Moscow State University.

### 2.12. Elemental Analysis of Fiber Coatings (EDS Analysis)

Elemental analysis was performed for PVP-PEG-1, PVP-PEG-2, PVP-PEG-1-Ag1, PVP-PEG-2-Ag1, PVP-PEG-1-Ag2, and PVP-PEG-2-Ag2 fiber samples using an energy-dispersive X-ray microanalyzer (EDS Oxford Instruments X-MAX-50, Oxford Instruments, Abingdon, UK) based on a VEGA3-LMU TESCAN scanning electron microscope (TESCAN, Brno, Czech Republic). The analytical study was carried out at the Department of Chemistry and Technology of Crystals MUCTR.

### 2.13. Determination of Antibacterial Activity by the Kirby-Bauer Method

The antibacterial activities of PVP-PEG-1, PVP-PEG-2, PVP-PEG-1-Ag1, PVP-PEG-2-Ag1, PVP-PEG-1-Ag2, and PVP-PEG-2-Ag2 were determined via the Kirby–Bauer disk diffusion method. To test for antibacterial activity, LB agar medium was added to 90 mm diameter Petri dishes, onto the surface of which a layer of the studied pathogenic bacteria (*Escherichia coli* and *Staphylococcus aureus*) was inoculated. During the experiment, 0.1 mL of the microorganism suspension at a concentration of 1.5 × 10^9^ cells ml^−1^ in the active growth phase was added and evenly distributed over the surface of the medium with a sterile spatula. Filter discs preimpregnated with the studied compounds were then added to the surface of the medium. Filter discs pre-soaked in LB medium served as a negative control. Cultivation was carried out at 37 °C for 24 h. The analytical study was performed at the Department of Biotechnology of the MUCTR. The antibacterial activity analysis revealed an inhibition zone of pathogenic bacteria, i.e., a zone free of microorganisms, the thickness of which was determined via ImageJ (V. 1.53k) [35].

Similar studies of antibacterial activity were conducted on the resulting fiber materials using the developed formulations. Instead of filter discs impregnated with the original formulations, fiber samples were added to the surface of the nutrient medium.

### 2.14. Methodology for Determining MIC and MBC

**Figure 3:**
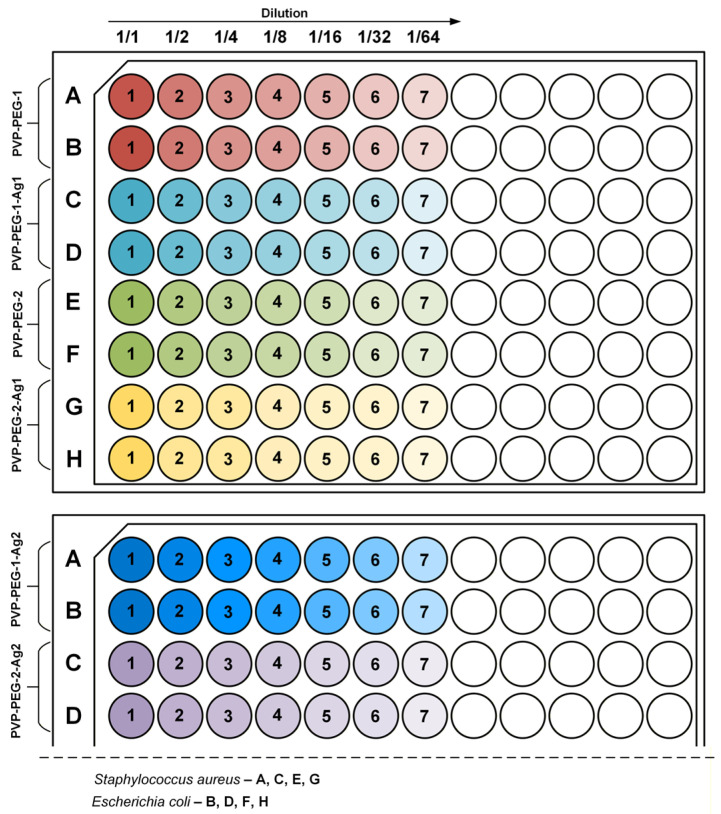
Methodology for determining MIC and MBC.

To quantitatively evaluate the antibacterial activity of the developed PVP-PEG formulations with silver nanoparticles, the minimum inhibitory and biocidal concentrations (MIC and MBC, respectively) were determined using dilution antimicrobial susceptibility test. Pathogenic bacteria used for the study were *Escherichia coli* and *Staphylococcus aureus*, grown in LB medium at 37 °C. The cultures of the test microorganisms were standardized by adjusting their optical density to values corresponding to the McFarland turbidity standard of 0.5 (equivalent to ~1 × 10^8^ cells/mL) through dilution with fresh sterile growth medium. The resulting standardized bacterial suspensions were used to inoculate 96-well culture plates, each well of which contained 150 μL of LB medium supplemented with one of the test formulations. Serial dilutions of the test formulations were carried out by sequentially diluting their concentrates with LB medium. The inoculated plates were incubated for 24 h on an orbital shaker at 350 rpm and 37 °C in an air-filled thermostat. Figure 3 shows a schematic diagram of the arrangement of the test compounds in the culture plates.

After completion of cultivation, MIC determinations were performed by visually assessing the growth of pathogen cultures. The MIC was defined as the minimum concentration of the biocidal agent at which no visible growth of microorganisms was observed. The MBC was defined as the minimum concentration of the biocidal agent in a well from which no colonies of the test culture formed when seeded. The analytical study was performed at the Department of Biotechnology of the MUCTR.

## 3. Results

### 3.1. Characteristics of PVP-PEG and PVP-PEG Solutions with Embedded AgNPs

**Figure 4:**
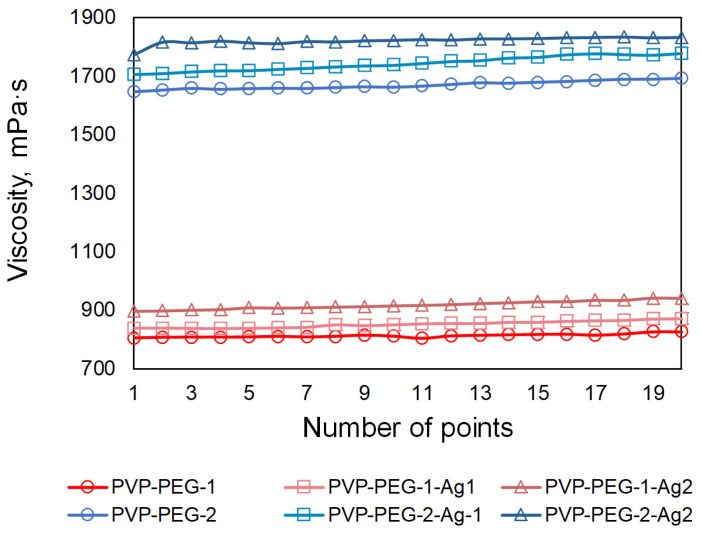
Viscosity values of the studied PVP-PEG-based compositions.

Figure 4 shows the results of the viscosity measurements for the PVP-PEG and PVP-PEG compositions with embedded AgNPs.

An analysis of the results revealed that increasing the PEG content from 3.5 wt.% to 7.5 wt.%, while maintaining the same PVP concentration in the studied solutions significantly increased the viscosity of the formulations, from 812.65 mPa·s to 1669.72 mPa·s for the PVP-PEG-1 and PVP-PEG-2 samples, respectively. This phenomenon is due to the increasing prevalence of intermolecular interaction forces. At a certain concentration, the regions of the polymer molecules begin to overlap, leading to entanglement of the polymer chains, which impedes flow and increases viscosity.

Increasing the molar concentration of AgNO_3_ from 0.002 M to 0.004 M in the PVP-PEG formulations promoted an increase in the concentration of AgNPs formed due to the reduction in AgNO_3_. An increase in the concentration of AgNPs also led to an increase in the viscosity of all the studied formulations. For the PVP-PEG-1-Ag1 and PVP-PEG-1-Ag2 solutions with a PEG content of 3.5 wt.%, the viscosity values were 851.52 and 917.68 mPa·s, respectively. For PVP-PEG-2-Ag1 and PVP-PEG-2-Ag2 solutions with a PEG content of 7.5 wt.%, the viscosity values also increased and were 1744.62 and 1820.77 mPa·s, respectively.

**Figure 5:**
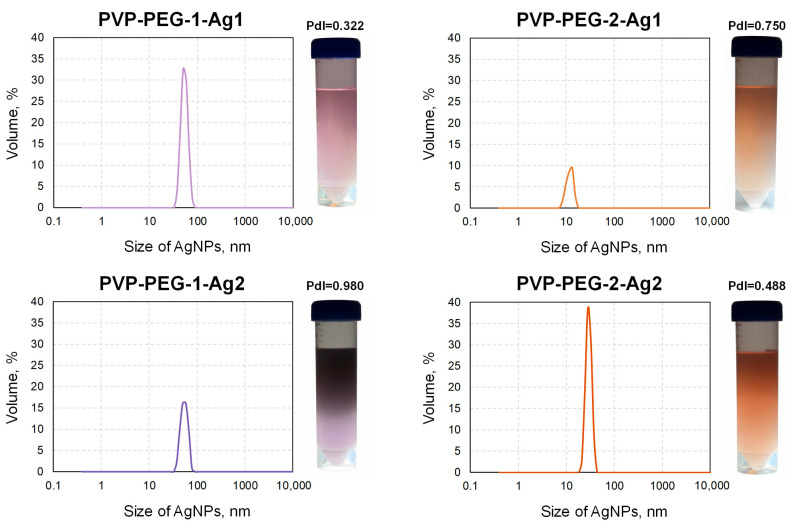
Results of measuring the diameters of the AgNPs in the studied PVP-PEG compositions.

The diameters of the synthesized AgNPs were determined for PVP-PEG-1-Ag1, PVP-PEG-2-Ag1, PVP-PEG-1-Ag2 and PVP-PEG-2-Ag2 solutions. The results are shown in Figure 5.

All the studied solutions of PVP-PEG-1-Ag1, PVP-PEG-2-Ag1, PVP-PEG-1-Ag2 and PVP-PEG-2-Ag2 have a monomodal size distribution of AgNPs. For the PVP-PEG-1-Ag1 and PVP-PEG-1-Ag2 samples, the peaks indicate the presence of AgNPs with characteristic sizes ranging from 37.84 to 78.82 nm. The color of the PVP-PEG-1-Ag1 and PVP-PEG-1-Ag2 solutions changes with increasing molar concentration of AgNO_3_ from light pink to dark purple. With an increase in the concentration of PEG, which acts as a reducing agent for AgNO_3_, the solutions of PVP-PEG-2-Ag1 and PVP-PEG-2-Ag2 had a dark orange color. The color of the solutions may depend on both the size and shape of the nanoparticles and their concentration [36]. The PVP-PEG-2-Ag1 sample has a peak in the range from 7.53 to 18.17 nm, while the PVP-PEG-2-Ag2 sample has a peak in the range from 21.04 to 43.82 nm. A higher PEG concentration (7.5 wt.%) led to a decrease in AgNPs size. Compared to the PVP-PEG-1-Ag1 and PVP-PEG-1-Ag2 samples with 3.5 wt.% PEG, the average AgNPs size for the PVP-PEG-2-Ag1 and PVP-PEG-2-Ag2 samples (7.5 wt.% PEG) decreased by 45.48 nm and 25.9 nm, respectively. A higher concentration of reducing agent (PEG) usually leads to an increase in nucleation sites and the formation of smaller AgNPs [37]. At the same time, the size of AgNPs is smaller for the PVP-PEG-2-Ag1 composition than for PVP-PEG-2-Ag2, which may be related to the higher molar concentration of AgNO_3_ in PVP-PEG-2-Ag2. At higher AgNO_3_ concentrations, more metal ions are available per reduction event, which promotes the growth of existing particles instead of the formation of new ones, resulting in an increase in the average size of the nanoparticles.

The polydispersity indices (Pdl) were 0.322, 0.750, 0.980 and 0.488 for the PVP-PEG-1-Ag1, PVP-PEG-2-Ag1, PVP-PEG-1-Ag2 and PVP-PEG-2-Ag2 solutions, respectively, indicating the heterogeneous composition of the nanoparticles in the studied formulations.

Table 3 shows the values of the ζ-potential determined for PVP-PEG solutions with embedded AgNPs.

One study [38] indicated that AgNPs with a ζ-potential in the range of −10 to +10 mV are considered neutral. However, nanoparticles with ζ-potential values greater than +10 mV or less than −10 mV are considered stable. The highest ζ-potential value (−15.5 mV) was observed for the PVP-PEG-2-Ag2 sample; therefore, this sample is the most stable of all those studied. However, precipitation was not observed for any of the studied samples during the observed month.

**Figure 6:**
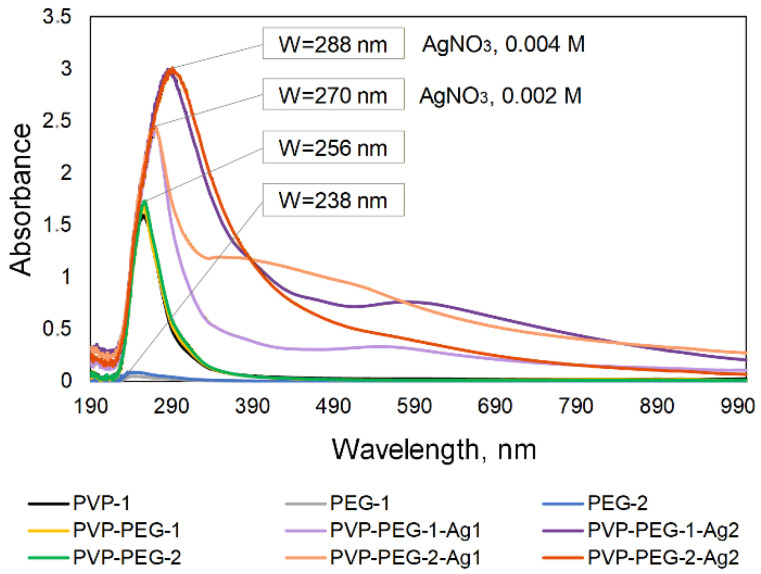
UV spectroscopy results.

The UV spectroscopy results revealed that the plasmon resonance peak in the 400–430 nm region, which is characteristic of AgNPs, is blurred (Figure 6).

With increasing particle size (>80 nm), scattering processes, rather than absorption, begin to dominate the spectrum. The plasmon resonance peak broadens significantly and shifts toward longer wavelengths. Instead of a sharp peak, a rise in the baseline is observed throughout the visible range. This result was confirmed by a study [39], which revealed that increasing the AgNPs size (~100 nm) led to the formation of a broader absorption peak without a clearly defined maximum in the UV spectra.

The presence of AgNPs in the PVP-PEG-1-Ag1, PVP-PEG-2-Ag1, PVP-PEG-1-Ag2 and PVP-PEG-2-Ag2 solutions was confirmed visually by color. Pink, dark purple, and orange are typical colors for silver sols of different sizes [40]. The presence of color indicates absorption in the visible region (400–700 nm), but it is represented not by a narrow peak, but by a broad band, which is observed as an increase in the curve in the spectrum (from 350 nm).

Peaks in the 238–256 nm region (PEG-1, PEG-2, PVP-1, PVP-PEG-1 and PVP-PEG-2 solutions) are associated with intramolecular electron transitions in polymers [41]. Upon the addition of AgNO_3_, the peaks shift to the 270–288 nm region (PVP-PEG-1-Ag1, PVP-PEG-2-Ag1, PVP-PEG-1-Ag2 and PVP-PEG-2-Ag2 solutions), with a significant increase in peak intensity, which is characteristic of the formation of Ag^+^ complexes with the PVP carbonyl group and PEG donor centers [42]. The highest peak intensity was achieved at an AgNO_3_ molar concentration of 0.004 M.

The results of measuring the electrical conductivity values of the solutions are presented in Table 4.

The electrical conductivity of solutions increases with increasing PEG concentration and increases significantly with increasing molar concentration of AgNO_3_.

### 3.2. Design and Assembly of a Portable Electrospinning Device

**Figure 7:**
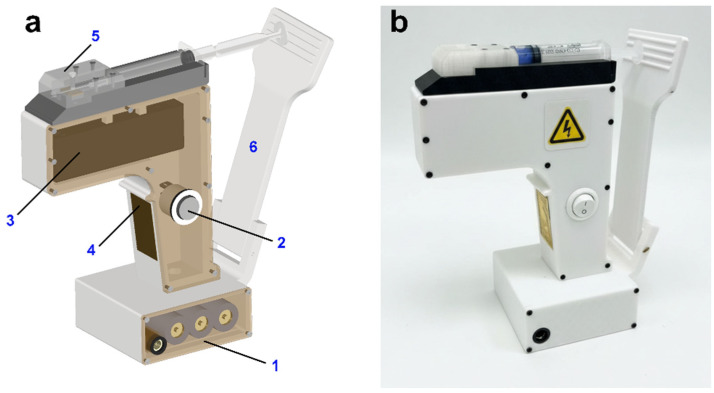
Portable electrospinning device: CAD model 1—battery, 2—power button, 3—high-voltage unit, 4—metal plate, 5—removable module with syringe, 6—syringe piston pressure regulator (**a**); appearance of the assembled device (**b**).

Figure 7 shows a computer-aided design (CAD) model of the developed design of a portable electrospinning device with the designation of the main working elements and the appearance of the assembled device.

The developed portable electrospinning device measures 18.7 × 13.3 × 3.5 cm and is equipped with a 12 V, 3500 m·Ah battery, providing over 2 h of continuous operation. The device’s characteristics allow it to be effectively used for prompt treatment of wounds with antibacterial compounds in the form of fiber polymer coatings.

Stable fiber formation is observed at a distance of 10–20 cm from the nozzle tip to the deposition surface, as confirmed by video recording of the electrospinning process (Video S1). Reducing the distance between electrodes (<10 cm) leads to solution dripping and the formation of defects on the already formed fiber layer.

To prevent short-circuiting of the electrical circuit, the portable electrospinning device is designed such that the needle tip (positive electrode) is recessed 0.5 cm deep inside the removable module (5).

When operating a portable electrospinning device, it is not recommended to reduce the distance between the two electrodes to less than 2 cm to avoid the formation of an ionized arc.

The advantages of the developed portable electrospinning device include: compact dimensions; ergonomic housing; the ability to charge the battery (the design features a dedicated connector for recharging); the high-voltage unit allows adjustment of the output voltage from 15 to 20 kV; the device housing is disassemblable, facilitating user replacement of individual components when needed; the syringe module (5) is removable and can be replaced with a dual-syringe module if required, enabling increased device productivity and application of fibers of various compositions to wounds.

The most significant drawback of the device is the manual extrusion of the polymer solution using the extrusion regulator (6). However, this drawback can be eliminated by adding a special carriage and a speed control circuit for its movement, which will automate the syringe piston displacement and, accordingly, the flow rate.

### 3.3. Structure of the Fiber Coatings

**Figure 8:**
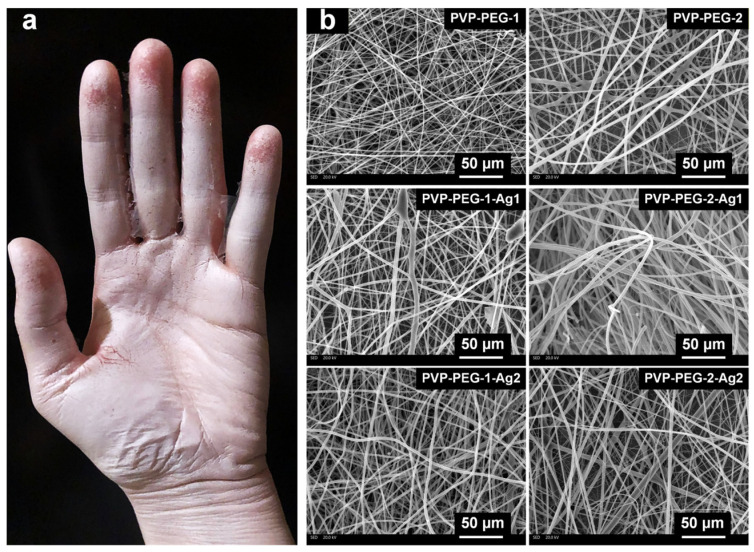
Application of PVP-PEG-based fibers to the skin: sample PVP-PEG-1 (**a**); SEM images (**b**).

Using the developed portable electrospinning device, PVP-PEG and PVP-PEG with AgNPs-based formulations were coated onto the skin to confirm the performance of the device and demonstrate the coating method (Figure 8a). To study the structure, the fibers were applied to the foil, after which scanning electron microscopy (SEM) images were obtained to analyze the morphology of the samples (Figure 8b).

All SEM images were processed in ImageJ, which was used to measure the diameters of the obtained fibers. For the measured data (Figure 8b), the compliance of the samples with a normal distribution was checked via the Shapiro–Wilk test [43]. Each sample included 30 measured objects to ensure data comparability:

(1) $$W=\frac{1}{s^{2}}{\left[\sum_{i=1}^{k}a_{n-i+1}\cdot \left(x_{n-i+1}-x_{i}\right)\right]}^{2}$$

(2) $$s^{2}=\sum_{i=1}^{N}{\left(x_{i}-\bar{x}\right)}^{2}$$

where *W* is the Shapiro–Wilk criterion; *s*^2^ is the sample variance, μm^2^; *x_i_* is the sample element, μm; $\bar{x}$ is the arithmetic mean, μm; $a_{n-i+1}$ are the coefficients determined from [43].

Null hypothesis *H*_0_: the random variable is normally distributed. Alternative hypothesis *H*_1_: the random variable is not normally distributed. The criterion for rejecting the null hypothesis is *W* < *W*(*p*). The critical value of the statistic for *p* = 0.05 is taken from [43].

For all the studied samples, the average diameter of the fibers was calculated via the following equation:

(3) $${\bar{d}}_{n}=\sum_{i}\frac{n_{i}}{\sum_{i}n_{i}}d_{i}$$

where ${\bar{d}}_{n}$ is the average diameter, μm; *n_i_* is the number of fibers in the *i*-th fraction; *d_i_* is the diameter of fibers in the *i*-th fraction, μm; $\sum_{i}n_{i}$ is the total number of measured fibers; $\frac{n_{i}}{\sum_{i}n_{i}}$ is the numerical proportion of fibers in the *i*-th fraction.

The results of the test of sample compliance with the normal distribution are summarized in Table 5.

The results show that the average diameter of the fibers increased linearly in the PVP-PEG-1, PVP-PEG-1-Ag1, PVP-PEG-1-Ag2 samples, as well as in the PVP-PEG-2, PVP-PEG-2-Ag1, and PVP-PEG-2-Ag2 samples, which is associated with the presence of AgNPs in the compositions, which linearly increased the viscosity of the initial solutions. Moreover, in the second group of samples, the average number diameter was greater than that in the first group, since the highest viscosity values were obtained for a PEG concentration of 7.5 wt.% (1669.72 mPa·s) rather than for a PEG concentration of 3.5 wt.% (812.65 mPa·s).

On the basis of the calculated Shapiro–Wilk criterion (Table 5), the following fiber samples were identified as having a normal distribution: PVP-PEG-2, PVP-PEG-1-Ag2, PVP-PEG-2-Ag1, and PVP-PEG-2-Ag2. The following viscosity values correspond to these samples: 1669.72, 917.67, 1744.62 and 1820.78 mPa·s. The viscosity values of samples PVP-PEG-1 and PVP-PEG-1-Ag1, which are 812.65 and 851.52 mPa·s, respectively, are outside the normal distribution. The viscosity of solutions greatly affects the fiber structure; insufficient flow properties can lead to unstable jet formation during electrospinning and, as a consequence, to an abnormal distribution of diameters by size. Moreover, samples with AgNPs were better suited for electrospinning, which is associated with the increased electrical conductivity of the compositions (Table 4).

**Figure 9:**
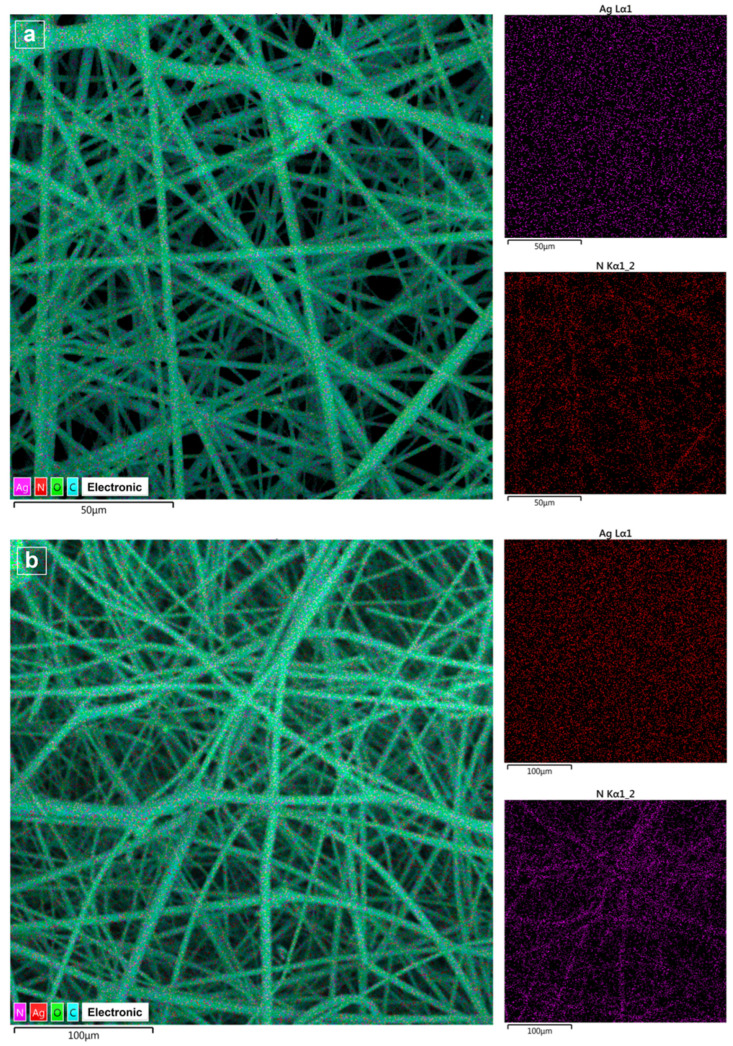
Surface mapping: PVP-PEG-2-Ag1 (**a**); PVP-PEG-2-Ag2 (**b**).

EDS analysis was performed to confirm the presence of silver in the fibers. As an example, Figure 9 shows the surface mapping of the PVP-PEG-2-Ag1 and PVP-PEG-2-Ag2 samples, reflecting the silver content. Surface mapping results for the original PVP-PEG-1, PVP-PEG-2 samples and the PVP-PEG-1-Ag1, PVP-PEG-1-Ag2 samples are presented in Supplementary Materials—Figure S1 and Figure S2, respectively.

EDS analysis data for PVP-PEG-1-Ag1 and PVP-PEG-1-Ag2 (Figure S2), PVP-PEG-2-Ag1 and PVP-PEG-2-Ag2 (Figure 9) samples showed that the silver integrated into the fibers was uniformly distributed.

The developed fiber polymer coatings could potentially protect the wound area from infection due to their antibacterial properties, high gas permeability due to their mesh structure, conformability to skin contours, and ease of removal or absorption of the compositions over time.

### 3.4. Antibacterial Activity of the Developed Compositions

**Figure 10:**
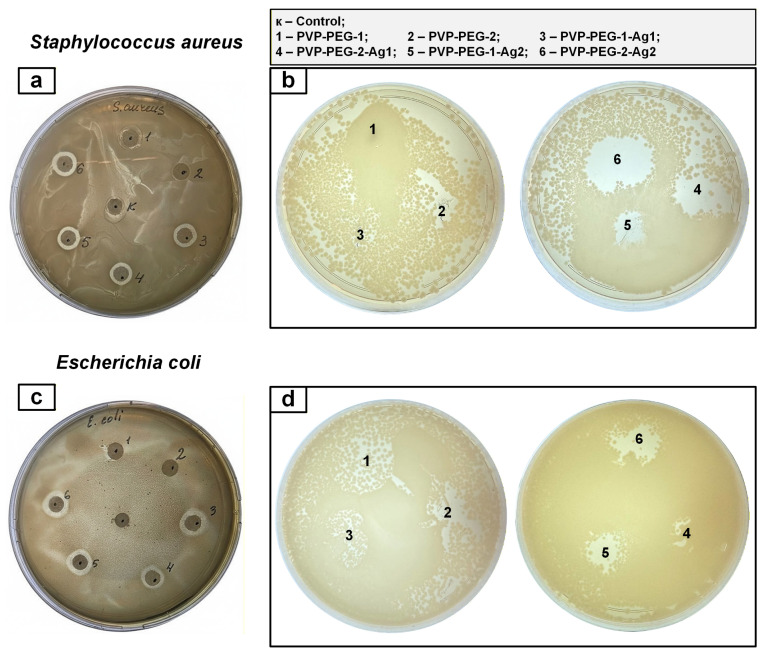
Results of the test of the studied compositions for antibacterial activity using the Kirby-Bauer method: diffusion disks (**a**,**c**); fibers (**b**,**d**).

Analysis of the results of the microbiological study revealed that samples modified with AgNPs exhibited antimicrobial activity against the studied pathogenic bacteria: *Escherichia coli* and *Staphylococcus aureus* (Figure 10).

The average inhibition zone thickness was calculated from three replicates for each pathogenic culture, with diameter measurements taken three times for each sample (Figure 10a,c). The inhibition zone calculation results and standard deviations (SD) are presented in Table 6.

The highest inhibition zone values were observed for samples against Gram-negative *Escherichia coli* bacteria, while Gram-positive *Staphylococcus aureus* bacteria were less sensitive to silver-containing samples (Figure 10a,c). This may be due to the different surface areas of the bacteria. Gram-negative bacteria have thinner cell membranes (8–12 nm) than Gram-positive bacteria (20–80 nm) [44]. The presence of a negatively charged lipopolysaccharide layer in Gram-negative bacteria promotes nanoparticle adhesion. These results are confirmed by a study [45], which also demonstrated a more effective antimicrobial effect of AgNPs-containing formulations against *Escherichia coli*.

All samples containing AgNPs exhibited resistance to pathogenic bacteria such as *Staphylococcus aureus* and *Escherichia coli*, which can cause purulent-inflammatory complications in wounds. Fiber samples (Figure 10b,d) also demonstrated inhibitory activity against *Staphylococcus aureus* and *Escherichia coli*; however, inhibition zone diameters were determined only using diffusion disks impregnated with the original compounds.

**Figure 11:**
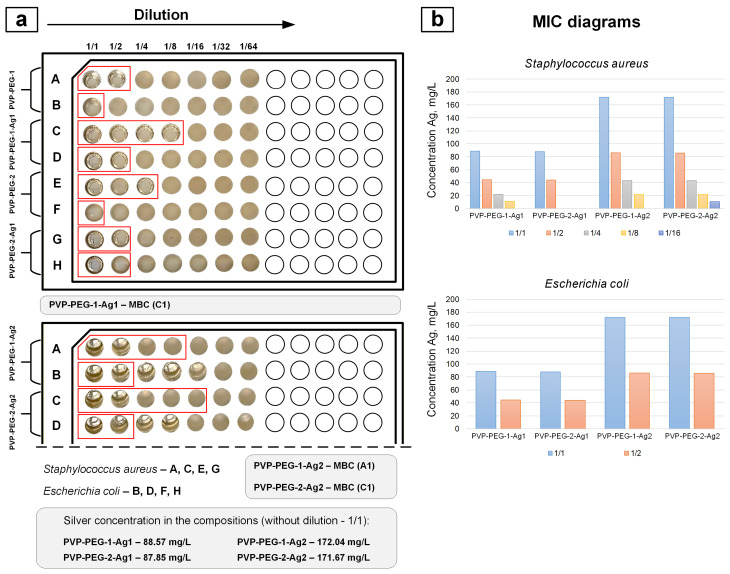
Results of MIC (samples are shown inside red frames) and MBC determination: serial dilution method (**a**); MIC diagrams (**b**).

Figure 11 shows the results of determining the MIC and MBC for the studied compounds.

The ICP-OES method was used to determine the silver concentrations in the initial formulations PVP-PEG-1-Ag1 (88.57 mg/L), PVP-PEG-2-Ag1 (87.85 mg/L), PVP-PEG-1-Ag2 (172.04 mg/L) and PVP-PEG-2-Ag2 (171.67 mg/L). Serial dilution of the formulations allowed us to determine the MIC in relation to *Staphylococcus aureus*: for the PVP-PEG-1-Ag1 sample, the MIC appears at dilution to 1/8 (11.07 mg/L); for the PVP-PEG-2-Ag1 sample, the MIC appears at dilution to 1/2 (43.92 mg/L); for the PVP-PEG-1-Ag2 sample, the MIC appears at dilution to 1/8 (21.51 mg/L); for the PVP-PEG-2-Ag2 sample, the MIC appears at dilution to 1/16 (10.73 mg/L). MICs against *Escherichia coli* were determined similarly. For all formulations, MICs were observed at dilutions up to 1/2: PVP-PEG-1-Ag1 (44.29 mg/L), PVP-PEG-2-Ag1 (43.93 mg/L), PVP-PEG-1-Ag2 (86.02 mg/L) and PVP-PEG-2-Ag2 (85.84 mg/L). The obtained MIC values against *Staphylococcus aureus* and *Escherichia coli* are consistent with literature data. In article [44], the MIC value was 13.5 mg/L AgNPs. In the same study, it was noted that subsequent development of resistance in *Staphylococcus aureus* increased the MIC to 54 mg/L. Such a silver concentration poses a toxic threat to mammalian cells. In work [46], a table is presented reflecting the toxicity threshold of AgNPs of different sizes with respect to various cell lines. It is indicated that for AgNPs obtained by chemical reduction method, with a size of 30–50 nm, the toxicity threshold occurs at a concentration of >50 μg/mL (50 mg/L) for A431 and A549 cells. For HT-1080 cells, the toxicity threshold occurs at a concentration of 6.25 μg/mL (6.25 mg/L) AgNPs with a size of 7–20 nm. In article [47], the MIC value against *Escherichia coli* was 85 μg/mL (85 mg/L).

It should be noted that MIC was also observed for silver-free formulations. This is due to the presence of PEG in the formulations, as it can exhibit antibacterial activity on its own.

The MBC values for the PVP-PEG-1-Ag1, PVP-PEG-1-Ag2, and PVP-PEG-2-Ag2 samples were 88.57 mg/L, 172.04 mg/L, and 171.67 mg/L, respectively. This means that the MBC was observed only for the original formulations without dilution. The MBC was not achieved for the PVP-PEG-2-Ag1 sample. It should be noted that the PVP-PEG-2-Ag1 sample had the lowest silver concentration in the studied series—87.85 mg/L. Therefore, it can be concluded that to achieve the MBC, the silver concentration in these formulations must be higher than this value.

The resulting compositions in the form of PVP-PEG fibers containing AgNPs exhibit antibacterial properties and can potentially be used as antibacterial wound dressings, providing complete adhesion to the skin and protection from bacteria.

## 4. Discussion

This article presents the main results of the development of an antibacterial coating based on PVP-PEG fibers with AgNPs and a method for applying these fibers to the skin. In the course of the study, compositions with various PEG concentrations of 3.5 and 7.5 wt% and AgNO_3_ molar concentrations of 0.002 and 0.004 M were obtained. AgNPs were synthesized in excess of PEG, which simultaneously acts as a reducing agent for Ag^+^ ions and a stabilizer of the colloidal system. The resulting silver sols had pink, dark purple, and orange colors, whereas the resulting nanoparticle size distributions were monomodal, with characteristic peaks of 50.75, 58.73, 13.54 and 28.21 nm for PVP-PEG-1-Ag1, PVP-PEG-1-Ag2, PVP-PEG-2-Ag1 and PVP-PEG-2-Ag2 respectively. The highest ζ-potential value for the AgNPs (sample PVP-PEG-2-Ag2) was –15.5 mV, indicating their stability. UV spectroscopy revealed that the plasmon resonance peak broadens significantly and shifts toward longer wavelengths, as scattering rather than absorption processes begin to predominate in the spectrum as the particle size increases (>80 nm).

The antibacterial activity of the formulations was tested against the pathogenic bacteria *Escherichia coli* and *Staphylococcus aureus*, which are the main causative agents of purulent-inflammatory processes in the wound area. The inhibition zone diameters for the PVP-PEG formulations with AgNPs were 8.4–8.9 mm (for *Staphylococcus aureus*) and 9.2–9.7 mm (for *Escherichia coli*). The developed formulations exhibit resistance to pathogenic biofilms and may be effective in combating resistance.

To form PVP-PEG-based fibers with antibacterial properties, a portable electrospinning device was developed at the Mendeleyev University of Chemical Technology of Russia. In the future, this device can be used for the prompt treatment of the wound area with antibacterial compounds in the form of fibrous polymer coatings, while providing protection and gas permeability to the wound area due to the dense mesh structure of the material. Analysis of the fiber coating structure revealed that the number-average diameter of the fibers is linearly dependent on the viscosity of the initial solutions. The study revealed working viscosity ranges of 1669.72, 917.67, 1744.62, and 1820.78 mPa·s, corresponding to samples of PVP-PEG-2, PVP-PEG-1-Ag2, PVP-PEG-2-Ag1, and PVP-PEG-2-Ag2, respectively. For these viscosity values, the diameter size distribution falls within the normal range.

This study confirmed the antibacterial activity of the developed PVP-PEG-based compositions containing silver nanoparticles, and also developed a method for applying the compositions to the skin as fiber coatings. Future work is planned to test the cytotoxicity and biocompatibility of the resulting fiber materials to evaluate their effectiveness as wound dressings.

## Figures and Tables

**Table 1:** Specification of PVP and PEG compositions.

Samples	Compositions		
	PVP, wt.%	PEG, wt.%	Solvent
PVP-1	12	–	IPA/Water (60:40)
PEG-1	–	3.5	
PEG-2	–	7.5	
PVP-PEG-1	12	3.5	
PVP-PEG-2	12	7.5	

**Table 2:** Specification of the studied PVP-PEG compositions with embedded AgNPs.

Samples	Compositions			
	PVP, wt.%	PEG, wt.%	AgNO_3_, M	Solvent
PVP-PEG-1-Ag1	12	3.5	0.002	IPA/Water (60:40)
PVP-PEG-2-Ag1		7.5		
PVP-PEG-1-Ag2		3.5	0.004	
PVP-PEG-2-Ag2		7.5		

**Table 3:** ζ-potential values for PVP-PEG solutions with embedded AgNPs.

Samples	ζ-Potential, mV
PVP-PEG-1-Ag1	−4.92
PVP-PEG-2-Ag1	−2.40
PVP-PEG-1-Ag2	−2.84
PVP-PEG-2-Ag2	−15.5

**Table 4:** Electrical conductivity of the solutions.

Samples	Electrical Conductivity, μS/cm
PVP-PEG-1	22
PVP-PEG-2	26
PVP-PEG-1-Ag1	37
PVP-PEG-2-Ag1	76
PVP-PEG-1-Ag2	56
PVP-PEG-2-Ag2	84

**Table 5:** Testing the compliance of a sample of PVP-PEG-based samples with a normal distribution.

Samples	${\bar{d}}_{n}$	*W*	*W*(*p*)	Hypothesis
PVP-PEG-1	1.03	0.899	0.927	*H*_1_
PVP-PEG-2	1.52	0.940		*H*_0_
PVP-PEG-1-Ag1	1.16	0.900		*H*_1_
PVP-PEG-2-Ag1	1.68	0.939		*H*_0_
PVP-PEG-1-Ag2	1.37	0.965		*H*_0_
PVP-PEG-2-Ag2	1.80	0.972		*H*_0_

**Table 6:** Inhibition zone thickness.

Samples	*Escherichia coli*		*Staphylococcus aureus*	
	Diameter, mm	SD, mm	Diameter, mm	SD, mm
PVP-PEG-1	–	–	–	–
PVP-PEG-2	–	–	–	–
PVP-PEG-1-Ag1	9.6	0.96	8.9	0.91
PVP-PEG-2-Ag1	9.2	0.59	8.4	0.90
PVP-PEG-1-Ag2	9.7	0.5	8.4	0.72
PVP-PEG-2-Ag2	9.3	0.65	8.8	0.56
Control	–	–	–	–

## Data Availability

The authors confirm that the data supporting the findings of this study are available within the article.

## Supplementary Materials

The following supporting information can be downloaded at: https://www.mdpi.com/article/10.3390/polym18091117/s1, Figure S1: surface mapping: PVP-PEG-1 (a); PVP-PEG-2 (b); Figure S2: surface mapping: PVP-PEG-1-Ag1 (a); PVP-PEG-1-Ag2 (b); Video S1: Fiber deposition using the developed portable electrospinning device.

## Abbreviations

The following abbreviations are used in this manuscript:

MUCTR	Mendeleev University of Chemical Technology of Russia
PVP	Polyvinylpyrrolidone
PEG	Polyethylene glycol
AgNO_3_	Silver nitrate
AgNPs	Silver nanoparticles
EDS	Energy-dispersive X-ray spectroscopy
MIC	Minimum inhibitory concentration
MBC	Minimum Bactericidal Concentration
Pdl	Polydispersity indices
SD	Standard deviations
CAD	Computer-aided design
SEM	Scanning electron microscopy

## References

[1] Petrosyan A., Martins P.N., Solez K., Uygun B.E., Gorantla V.S., Orlando G. (2022). Regenerative medicine applications: An overview of clinical trials. Front. Bioeng. Biotechnol..

[2] Wang J., Deng G., Wang S., Li S., Song P., Lin K., Xu X., He Z. (2024). Enhancing regenerative medicine: The crucial role of stem cell therapy. Front. Neurosci..

[3] Iovene A., Zhao Y., Wang S., Amoako K. (2021). Bioactive Polymeric Materials for the Advancement of Regenerative Medicine. J. Funct. Biomater..

[4] Al-Khafaji Z., Brito S., Bin B.H. (2022). Zinc and Zinc Transporters in Dermatology. Int. J. Mol. Sci..

[5] Yousef H., Alhajj M., Sharma S. (2017). Anatomy, Skin (Integument), Epidermis. StatPearls.

[6] Harding K.G. (2022). Chronic wounds: A clinical problem requiring ownership and coordination. BJD.

[7] Markiewicz-Gospodarek A., Kozioł M., Tobiasz M., Baj J., Radzikowska-Büchner E., Przekora A. (2022). Burn Wound Healing: Clinical Complications, Medical Care, Treatment, and Dressing Types: The Current State of Knowledge for Clinical Practice. Int. J. Environ. Res. Public Health.

[8] Wang C., Sani E.S., Shih C.-D., Lim C.T., Wang J., Armstrong D.G., Gao W. (2024). Wound management materials and technologies from bench to bedside and beyond. Nat. Rev. Mater..

[9] Eriksson E., Liu P.Y., Schultz G.S., Martins-Green M.M., Tanaka R., Weir D., Gould L.J., Armstrong D.G., Gibbons G.W., Wolcott R. (2022). Chronic wounds: Treatment consensus. Wound Repair Regen..

[10] Pisani S., Tufail S., Rosalia M., Dorati R., Genta I., Chiesa E., Conti B. (2024). Antibiotic-Loaded Nano-Sized Delivery Systems: An Insight into Gentamicin and Vancomycin. J. Funct. Biomater..

[11] Ezike T.C., Okpala U.S., Onoja U.L., Nwike C.P., Ezeako E.C., Okpara O.J., Okoroafor C.C., Eze S.C., Kalu O.L., Odoh E.C. (2023). Advances in drug delivery systems, challenges and future directions. Heliyon.

[12] Boateng J.S., Matthews K.H., Stevens H.N., Eccleston G.M. (2008). Wound healing dressings and drug delivery systems: A review. J. Pharm. Sci..

[13] Febriyenti F., Lucida H., Almahdy A., Alfikriyah I., Hanif M. (2019). Wound-healing effect of honey gel and film. J. Pharm. Bioallied Sci..

[14] Tijani A.O., Nunez E., Singh K., Khanna G., Puri A. (2021). Transdermal Route: A Viable Option for Systemic Delivery of Antidepressants. J. Pharm. Sci..

[15] Abramov E., Garti N. (2021). Development of polymeric films embedded with liquid nanodomains. J. Colloid Interface Sci..

[16] Jahani M., Asefnejad A., Al-Musawi M.H., Mohammed A.A., Al-Sudani B.T., Al-Bahrani M.H., Kadhim N.A., Shahriari-Khalaji M., Valizadeh H., Sharifianjazi F. (2024). Antibacterial and wound healing stimulant nanofibrous dressing consisting of soluplus and soy protein isolate loaded with mupirocin. Sci. Rep..

[17] Vasireddi R., Kruse J., Vakili M., Kulkarni S., Keller T.F., Monteiro D.C.F., Trebbin M. (2019). Solution blow spinning of polymer/nanocomposite micro-/nanofibers with tunable diameters and morphologies using a gas dynamic virtual nozzle. Sci. Rep..

[18] Sharma A. (2021). Rifampicin-Loaded Alginate-Gelatin Fibers Incorporated within Transdermal Films as a Fiber-in-Film System for Wound Healing Applications. Membranes.

[19] Alven S., Aderibigbe B.A. (2021). Fabrication of Hybrid Nanofibers from Biopolymers and Poly (Vinyl Alcohol)/Poly (ε-Caprolactone) for Wound Dressing Applications. Polymers.

[20] Lu X., Zhou L., Song W. (2024). Recent Progress of Electrospun Nanofiber Dressing in the Promotion of Wound Healing. Polymers.

[21] Medic B.S., Tomic N., Lagopati N., Gazouli M., Pojskic L. (2024). Advances in Metal and Metal Oxide Nanomaterials for Topical Antimicrobial Applications: Insights and Future Perspectives. Molecules.

[22] Mercan D.A., Niculescu A.G., Grumezescu A.M. (2022). Nanoparticles for Antimicrobial Agents Delivery—An Up-to-Date Review. Int. J. Mol. Sci..

[23] Ding X., Tang Q., Xu Z., Xu Y., Zhang H., Zheng D., Wang S., Tan Q., Maitz J., Maitz P.K. (2022). Challenges and innovations in treating chronic and acute wound infections: From basic science to clinical practice. Burn. Trauma.

[24] Mondal S.K., Chakraborty S., Manna S., Mandal S.M. (2024). Antimicrobial nanoparticles: Current landscape and future challenges. RSC Pharm..

[25] Chen H., Zhang H., Shen Y., Dai X., Wang X., Deng K., Long X., Liu L., Zhang X., Li Y. (2021). Instant in-situ Tissue Repair by Biodegradable PLA/Gelatin Nanofibrous Membrane Using a 3D Printed Handheld Electrospinning Device. Tissue Eng. Regen. Med..

[26] Yan X., Yu M., Ramakrishna S. (2019). Advances in portable electrospinning devices for in situ delivery of personalized wound care. Nanoscale.

[27] Liu S., Wu G., Wang W., Wang H., Gao Y., Yang X. (2023). In Situ Electrospinning of “Dry-Wet” Conversion Nanofiber Dressings for Wound Healing. Mar. Drugs.

[28] Brako F., Luo C., Craig D.Q.M., Edirisinghe M. (2018). An Inexpensive, Portable Device for Point-of-Need Generation of Silver-Nanoparticle Doped Cellulose Acetate Nanofibers for Advanced Wound Dressing. Macromol. Mater. Eng..

[29] Jin W.-J., Lee H.K., Jeong E.H., Park W.H., Youk J.H. (2005). Preparation of Polymer Nanofibers Containing Silver Nanoparticles by Using Poly(N-vinylpyrrolidone). Macromol. Rapid Commun..

[30] Huang S., Zhou L., Li M.-C., Wu Q., Kojima Y., Zhou D. (2016). Preparation and Properties of Electrospun Poly (Vinyl Pyrrolidone)/Cellulose Nanocrystal/Silver Nanoparticle Composite Fibers. Materials.

[31] Stoyanova N., Georgieva A., Toshkova R., Spasova M. (2026). Fabrication and Detailed Characterization of PLA/PEG Composite Nanofibers for the Co-Delivery and Synergistic Release of Quercetin and Rosmarinic Acid via Electrospinning. Molecules.

[32] Zhang W., Zhang X., Xu Y., Xu Y., Qiao J., Shi T., Huang Z., Liu Y., Fang M., Min X. (2021). Flexible polyethylene glycol/polyvinylpyrrolidone composite phase change fibres: Preparation, characterization, and thermal conductivity enhancement. Polymer.

[33] Luo C., Zhang Y., Zeng X., Zeng Y., Wang Y. (2005). The role of poly(ethylene glycol) in the formation of silver nanoparticles. J. Colloid Interface Sci..

[34] Dhakal T.R., Mishra S.R., Glenn Z., Rai B.K. (2012). Synergistic effect of PVP and PEG on the behavior of silver nanoparticle-polymer composites. J. Nanosci. Nanotechnol..

[35] Mokhova E., Gordienko M., Menshutina N., Kalenov S., Avetissov I., Eremeev A. (2023). Influence of Ultrasound on the Properties of Polysaccharide Complexes and Materials Based on Them. Polysaccharides.

[36] González A.L., Noguez C., Beránek J., Barnard A. (2014). Size, Shape, Stability, and Color of Plasmonic Silver Nanoparticles. J. Phys. Chem. C.

[37] Laib I., Gheraissa N., Benaissa A., Benkhira L., Azzi M., Benaissa Y., Abdelaziz A.G., Tian F., Walsh M., Bechelany M. (2025). Tailoring innovative silver nanoparticles for modern medicine: The importance of size and shape control and functional modifications. Mater. Today Bio.

[38] Abdellatif A.A.H., Alturki H., Tawfeek H.M. (2021). Different cellulosic polymers for synthesizing silver nanoparticles with antioxidant and antibacterial activities. Sci. Rep..

[39] De Leersnyder I., De Gelder L., Van Driessche I., Vermeir P. (2019). Revealing the Importance of Aging, Environment, Size and Stabilization Mechanisms on the Stability of Metal Nanoparticles: A Case Study for Silver Nanoparticles in a Minimally Defined and Complex Undefined Bacterial Growth Medium. Nanomaterials.

[40] Frank C.J., He V., Scaiano J.C., Silvero C.M.J. (2025). Photoinduced Transport and Activation of Polymer-Embedded Silver on Rice Husk Silica Nanoparticles for a Reusable Antimicrobial Surface. Nanomaterials.

[41] Laatsch B.F. (2023). Polyethylene Glycol 20k. Does It Fluoresce?. ACS Omega.

[42] Jayaramudu T. (2016). Preparation and characterization of poly(ethylene glycol) stabilized nano silver particles by a mechanochemical assisted ball mill process. J. Appl. Polym. Sci..

[43] Singh P., Pandit S., Jers C., Joshi A.S., Garnæs J., Mijakovic I. (2021). Silver nanoparticles produced from *Cedecea* sp. exhibit antibiofilm activity and remarkable stability. Sci. Rep..

[44] Hochvaldová L., Panáček D., Válková L., Večeřová R., Kolář M., Prucek R., Kvítek L., Panáček A. (2024). *E. coli* and *S. aureus* resist silver nanoparticles via an identical mechanism, but through different pathways. Commun. Biol..

[45] Shapiro S.S., Wilk M.B. (1965). An analysis of variance test for normality (complete samples). Biometrika.

[46] Akter M., Sikder M.T., Rahman M.M., Ullah A.K.M.A., Hossain K.F.B., Banik S., Hosokawa T., Saito T., Kurasaki M. (2018). A systematic review on silver nanoparticles-induced cytotoxicity: Physicochemical properties and perspectives. J. Adv. Res..

[47] Selem E., Mekky A.F., Hassanein W.A., Reda F.M., Selim Y.A. (2022). Antibacterial and antibiofilm effects of silver nanoparticles against the uropathogen Escherichia coli U12. Saudi J. Biol. Sci..

