# Supplementary figures and images for: Development of an Antibacterial Coating Based on PVP–PEG Fibers Incorporating Silver Nanoparticles and a Method for Its Application to Skin
Source: Polymers (Basel). 2026 Apr 30;18(9):1117. doi: 10.3390/polym18091117 (PMC13165970; doi:10.3390/polym18091117)

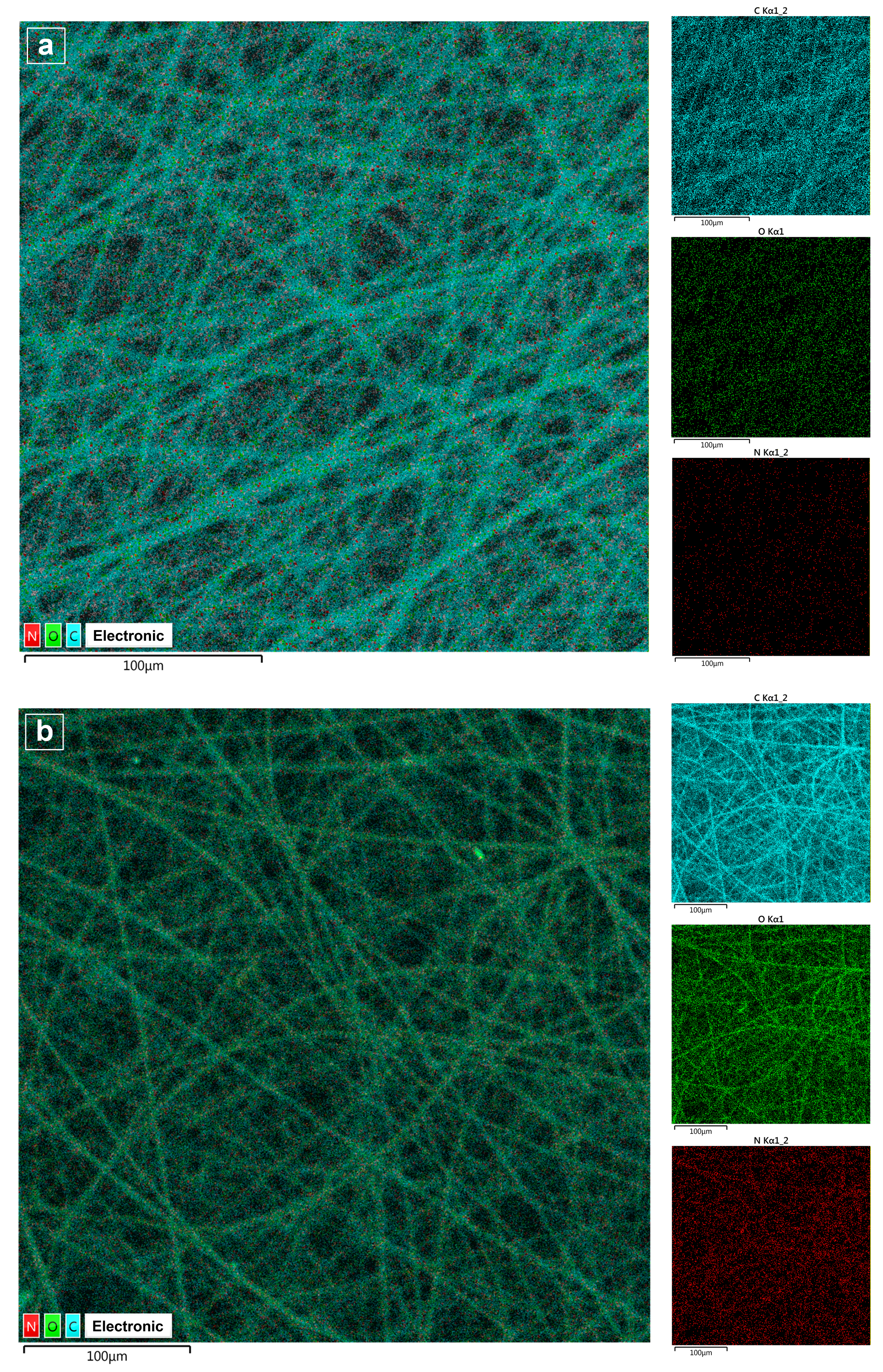

Supplement: Supplementary file 1 [file polymers-18-01117-s001.zip › Figure_S1.png]

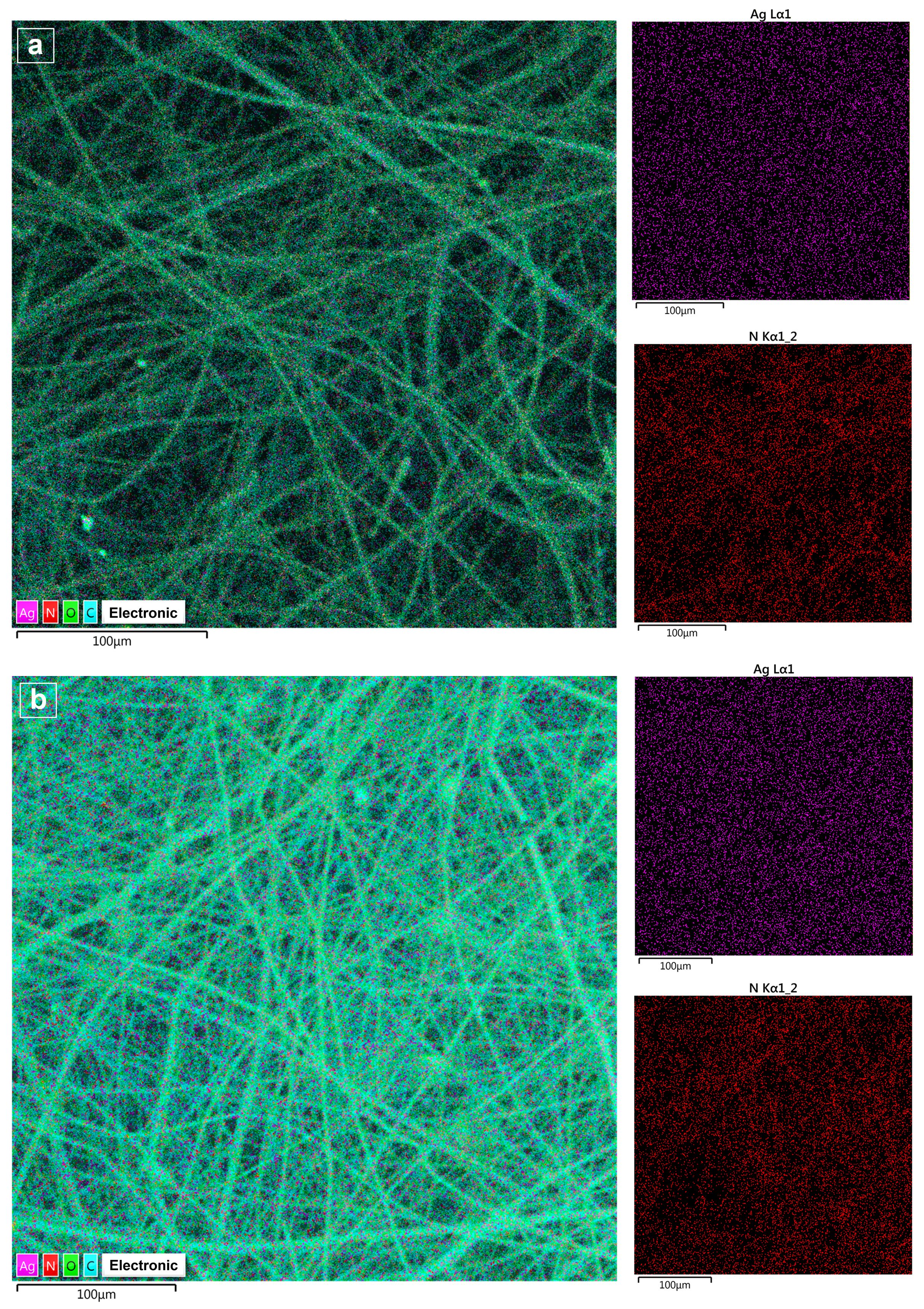

Supplement: Supplementary file 1 [file polymers-18-01117-s001.zip › Figure_S2.png]
